# STIM1 in tumor cell death: angel or devil?

**DOI:** 10.1038/s41420-023-01703-8

**Published:** 2023-11-06

**Authors:** Ran Ren, Yongsheng Li

**Affiliations:** 1 Chongqing University Cancer Hospital, School of Medicine, Chongqing University, 400044 Chongqing, China; 2 Department of Medical Oncology, Chongqing University Cancer Hospital, 400030 Chongqing, China

**Keywords:** Cancer, Molecular biology

## Abstract

Stromal interaction molecule 1 (STIM1) is involved in mediating the store-operated Ca^2+^ entry (SOCE), driving the influx of the intracellular second messenger calcium ion (Ca^2+^), which is closely associated with tumor cell proliferation, metastasis, apoptosis, autophagy, metabolism and immune processes. STIM1 is not only regulated at the transcriptional level by NF-κB and HIF-1, but also post-transcriptionally modified by miRNAs and degraded by ubiquitination. Recent studies have shown that STIM1 or Ca^2+^ signaling can regulate apoptosis, autophagy, pyroptosis, and ferroptosis in tumor cells and act discrepantly in different cancers. Furthermore, STIM1 contributes to resistance against antitumor therapy by influencing tumor cell death. Further investigation into the mechanisms through which STIM1 controls other forms of tumor cell death could aid in the discovery of novel therapeutic targets. Moreover, STIM1 has the ability to regulate immune cells within the tumor microenvironment. Here, we review the basic structure, function and regulation of STIM1, summarize the signaling pathways through which STIM1 regulates tumor cell death, and propose the prospects of antitumor therapy by targeting STIM1.

## Facts

- STIM1 is one of the major molecules that constitute CRAC, which is a channel that drives Ca^2+^ influx for SOCE function and is closely associated with tumor cell proliferation, metastasis, death, and immunity.
- STIM1 can be regulated at the transcriptional level by PI3K-SGK1-P300-NF-κB and CaMKKII-P300-HIF-1 signaling pathways, as well as post-transcriptionally modified by various miRNAs and also directly degraded by ubiquitination.
- Apoptosis, autophagy, pyroptosis, ferroptosis, and cuproptosis are classical forms of programmed cell death, while STIM1 has the ability to influence the occurrence of these cell deaths in tumor cells.
- STIM1 strengthens antitumor therapy by potentiating tumor cell death and antitumor immunity by enhancing the proliferation, activation, and memory of immune cells.
- The identification of effective new drugs that target STIM1 to enhance tumor cell death is a promising direction for future antitumor therapy.

## Open questions

- What are the various pathways through which STIM1 influences tumor cell death in different types of cancers?
- How can STIM1 regulate resistance to antitumor therapy and tumor immunity by affecting cell death?
- What are the potential future applications of targeting STIM1 for the treatment of human tumors?

## Introduction

Calcium ion (Ca^2+^) is a vital intracellular second messenger. A variety of cellular activities are controlled by changes of intracellular Ca^2+^ concentration, such as cell proliferation, apoptosis, metabolism, and immunity [1–4]. In nonexcitable cells such as tumor cells, the major route for the extracellular Ca^2+^ influx through the plasma membrane (PM) is the Ca^2+^ release-activated Ca^2+^ channel (CRAC), which mediates the store-operated Ca^2+^ entry (SOCE). The function of SOCE has been extensively studied, but its regulatory mechanisms remained a mystery until 2005 [5] and 2006 [6] when stromal interaction molecule 1 (STIM1) and Orai1 were identified as key molecules that maintain SOCE function. STIM1 is a transmembrane protein that spans the endoplasmic reticulum (ER) membrane, with its N-terminal anchored to the ER membrane and its C-terminal located in the cytosol [7], while Orai1 is a protein located on the PM that is highly selective for Ca^2+^ [8]. When the Ca^2+^ pool of the ER is depleted, STIM1 senses the signal and oligomerizes, followed by STIM1 translocation to the PM to activate Orai1 at the ER-PM junction, thereby activating CRAC and SOCE and causing Ca^2+^ influx [9]. In addition to activating Orai1, STIM1 can also activate Orai2 and Orai3 in mammals to produce I_CRAC_, although with less efficiency [10]. It is worth noting that the canonical TRP (TRPC) channels, along with STIM1 and Orai1, are involved in SOCE [11]. Guido et al. demonstrated that junctate, a single transmembrane protein located in the ER, can mediate SOCE by recruiting the ER junctions even in the absence of the STIM1 protein [12]. These findings suggest that SOCE can be triggered without specifically targeting the STIM1 protein, offering a new approach for cancer therapy.

Recently, it has become increasingly clear that STIM1 is involved in regulating the occurrence, development, invasion, and metastasis of a variety of cancers. In these processes, tumor cell death plays a crucial role. Cell death can be classified as accidental cell death (ACD) and regulated cell death (RCD) according to whether it is regulated by genes [13–15]. Recently, compelling evidence indicates the induction of tumor cell death can enhance the effects of antitumor immunotherapy [16–18], and that STIM1-induced tumor cell death can alleviate the resistance to antitumor therapy [19–21].

In this context, we review the discovery, structure, and activation process of STIM1, the upstream signaling pathways regulating STIM1, summarize the functions of STIM1 on tumor cell death and the pathways through which STIM1 reverses resistance to antitumor therapy by affecting tumor cell death, and the key role of STIM1 in tumor immunity. Finally, we discuss the clinical applications of STIM1 and future prospects.

## STIM1: discovery, structure, and activation

### Discovery of STIM1

In 2005, Parekh AB and Putney JW Jr proposed that four main types of Ca^2+^ channels exist in the cell membrane and the store-operated channels (SOC) is considered to be the most primitive pathway for Ca^2+^ entry into cells [22]. Although STIM was first discovered by Parker in 1996 [23], its functions were not clear. Roos identified STIM1 as the molecular basis for the composition of thapsigargin (TG)-dependent Ca^2+^ influx and CRAC channels, by suppressing multiple genes in *Drosophila* S2 cells by RNA interference (RNAi) [5].

### Structure of STIM1

**Fig. 1:**
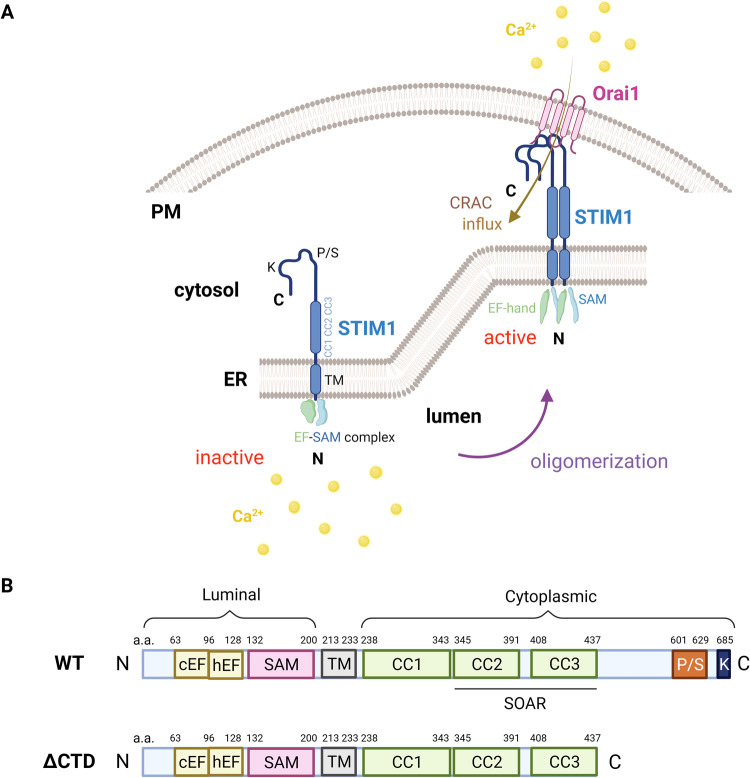
Location and structure of STIM1. **A** STIM1 is localized primarily in the ER membrane, with a small fraction in the PM. Orai1 is a plasma membrane protein with four membrane-spanning regions and intracellular N and C-terminus. When Ca^2+^ in the ER lumen is depleted, the EF-SAM complex disintegrates. Subsequently, STIM1 oligomerizes and translocates to the PM, then binds to Orai1 on the PM, which activates CRAC and causes Ca^2+^ influx. **B** The domains of STIM1 are predicted. Starting at the N-terminal, STIM1 includes a canonical EF-hand (nEF), a hidden EF-hand (hEF) and sterile-a motif (SAM) domains on the luminal side. On the cytosolic side are overlapping coiled-coil (CC) and serine–proline-rich (S/P) and lysine-rich (K) domains.

STIM encodes a dimeric type I transmembrane protein with two homologs in mammals, STIM1 and STIM2. STIM1 is located in the region of human chromosome 11p15.5 [23, 24]. STIM1 consists of three distinct components: the N-terminal located in the lumen of the endoplasmic reticulum (ER), the cytoplasmic chain, and the C-terminal that is soluble in the cytosol [25]. The N-terminal portion includes a canonical EF-hand (cEF), a hidden (hEF) EF-hand, and a sterile α motif (SAM). The cytoplasmic chain region is composed of three coiled-coil (CC) domains known as CC1, CC2, and CC3. CC1 and the SAM domain are connected by a transmembrane domain (TM). In addition, the STIM-Orai-activating region (SOAR), also referred to as the CRAC-activating domain (CAD), is comprised of fragments from CC2 and CC3. Besides, the C-terminal segment is composed of Pro/Ser-rich region and Lys-rich region [7, 26] (Fig. 1A). In the last decade, the STIM1 structural domain and its conformational changes during activation have been better understood by NMR spectroscopy and X-ray crystallography [26–29].

### Activation of STIM1

STIM1 performs two main functions: first, it has the ability to sense precise changes in the concentration of Ca^2+^ (100–400 μM) in the ER lumen [30], and second, it conveys this signal to Orai1 on PM and activates SOCE.

When cells are at rest condition, the cEF-hand of STIM1 is responsible for binding to ER luminal Ca^2+^ and the hEF-hand maintains the stability [31]. When G protein-coupled receptors (GPCRs) or protein tyrosine kinase-linked receptors (PTKRs) on the PM are activated by hormones and growth factors, they bind to the corresponding phospholipase C (PLC) isoforms, resulting in 1,4,5-trisphosphate (InsP3) and diacylglycerol (DAG), respectively. Subsequently, InsP3 binds to InsP3R on the ER membrane, causing the release of Ca^2+^ [2]. When the Ca^2+^ concentration in the ER lumen falls below 300 μM (Hill coefficient approximately equal to 4), it separates from the cEF-hand of STIM1, thereby destabilizing the EF-SAM complex and leading to STIM1 oligomerization [32]. Immediately afterward, STIM1 translocates to the ER-PM junction sites and physically interacts with Orai1, opening the CRAC channel and causing Ca^2+^ entry [30, 33]. Furthermore, reliable experiments have declared that the cytosolic C-terminus of STIM1 is the key domain for opening the Orai1 channel [34] (Fig. 1B). Through the induction of cellular mutations and subcellular localization, crucial functions of STIM1 in constituting CRAC channels and SOCE activation have been progressively elucidated.

### Isoforms of STIM1

To date, three splicing variant isoforms of STIM1 have been identified and studied. The first isoform, known as STIM1L, was discovered in 2011 and is specifically expressed in the brain and heart of mice, cardiomyocytes of neonatal rats, and skeletal muscle cells of humans. STIM1L is generated through alternative splicing of exon 11. Its C-terminal region, along with an additional 106 residues, forms a permanent actin-binding domain (ABD), resulting in a significant increase in the rate and frequency of SOCE activation [35]. The second isoform, referred to as STIM1A or STIM1β, was discovered in 2020 and is highly expressed in astrocytes, heart, kidney, and testis. It is formed by the insertion of an extra 31 amino acid residues at position 491 in the C-terminal of STIM1 [36]. Xie et al. found that STIM1β, which is upregulated in glioblastoma, can enhance the function of SOCE by acting as a potent activator of the Orai1 channel [37]. On the other hand, full-length STIM1A functions to negatively regulate SOCE and I_CRAC_, but it enhances NFAT translocation by reducing cAMP degradation [38]. Another variant, STIM1B, was discovered in 2021 and is highly abundant in the central nervous system (CNS). STIM1B is generated by inserting a specific motif into the cytoplasmic region of STIM1, which allows it to be specifically targeted. Experimental results have shown that STIM1B exhibits a slow recruitment to the ER-PM junctions, leading to a decrease in both the rate and peak of SOCE, as well as a reduction in I_CRAC_ [39].

## The upstream regulatory pathways of STIM1

**Fig. 2:**
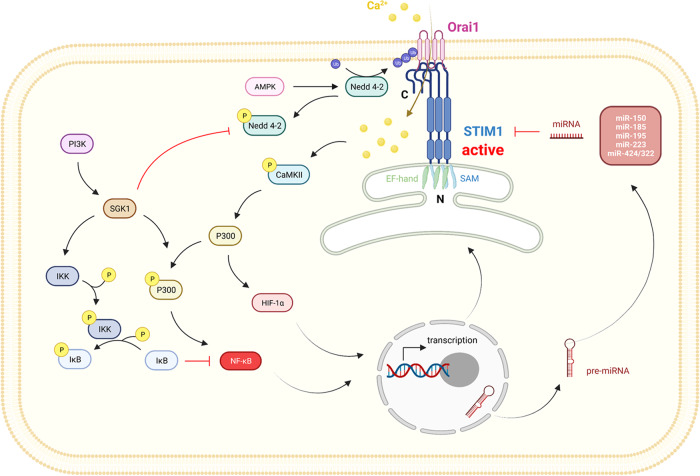
Upstream regulatory pathways of STIM1. The transcriptional levels of STIM1 and Orai1 can be modulated by multiple signaling pathways, including PI3K-SGK1-NF-κB and CaMKII-P300-HIF-1α axes. In addition, the ubiquitination of Orai1 can be triggered by AMPK-Nedd4-2. At the level of post-transcriptional modification, the expression levels of STIM1 in a variety of tumors are suppressed by a variety of miRNAs, such as miR-150, miR-185, miR-195, miR-223, and miR-424/322.

STIM1/Orai is the fundamental component of CRAC channels and plays an important role in the regulation of intracellular Ca^2+^ homeostasis mediated by SOCE. Various intracellular signals affect intracellular Ca^2+^ concentrations by regulating STIM1/Orai (Fig. 2).

For instance, transcription factor nuclear factor kappa B (NF-κB) has been shown to increase the mRNA and protein levels of STIM1 and Orai1 [40–42]. Further experiments demonstrated that the transcriptional promotion of STIM1 and Orai1 by NF-κB is achieved through the binding of p65 to the S1 and O2 regions of their promoters, respectively. Moreover, studies demonstrated that subunits p65/p50 or p65/p52 of NF-κB enhance the migratory capacity of HEK293 cells through transcriptional activation of STIM1 and Orai1 [43]. Our earlier study reported that hypoxia-inducible factor-1 (HIF-1) could transcript STIM1 to enhance the SOCE in hepatocellular carcinoma (HCC) cells [44].

In addition to regulating the transcriptional levels of STIM1 and Orai1, Keil found that Plenty of SH3’s (POSH), an E3 ubiquitin ligase, could directly ubiquitinate STIM1 and initiate its degradation, thereby reducing intracellular Ca^2+^ [45]. Similarly, studies have shown that neuronal precursor cells expressed developmentally downregulated 4-2 (Nedd4-2), also an E3 ubiquitin ligase, was able to ubiquitinate and degrade Orai1, resulting in Orai1 not being activated by STIM1 to perform SOCE functions [46]. In terms of regulating Nedd4-2, Almaca et al. found that AMPK could activate it [47, 48], and later Nurbaeva et al. also reported high expression of STIM1 and Orai1 in the dendritic cells (DCs) of AMPK-deficient mice [49].

Serum and glucocorticoid inducible kinase 1 (SGK1) are a downstream target of phosphatidylinositol 3-kinase (PI3K) signaling [50–52]. Experiments have shown that the transcription levels of STIM1 and Orai1 in SGK1-deficient mice were significantly lower than those in the control group [53]. In addition, SGK1 can phosphorylate Nedd4-2, thereby reversing the reduction the influx of Ca^2+^ [46]. Overall, SGK1 facilitates cell migration, mast cell degranulation, and platelet aggregation by up-regulating STIM1/Orai1 and thereby promoting SOCE function [54, 55].

MicroRNAs (miRNAs) are involved in post-transcriptional regulation of gene expression [56]. Recently, miRNAs have been revealed to regulate STIM1 in various tissues by post-transcriptionally modifying the 3’-untranslated region (3’-UTR) of STIM1 mRNA. On the one hand, in normal cells, such as in intestinal epithelial cells, miR-195 competes with the RNA-binding protein HUR to bind the 3’UTR of STIM1 mRNA, rendering it unstable and thus inhibiting cell migration [57]. In addition, in vascular smooth muscle cells (VSMC), miR-424/322 prevents VSMC proliferation and tissue repair by impairing STIM1 and cyclin D1 [58]. On the other hand, in cancer cells, such as colorectal cancer (CRC) cells, miR-185 blunts the expression of STIM1, thereby suppressing the metastasis and invasion of CRC [59]. Moreover, in luminal non-aggressive MCF7 breast cancer cells, miR-223 and miR-150 decline the expression of STIM1, but the impact of this regulation on breast cancer remains unexplored [60].

Taken together, the transcriptional, post-transcriptional modification, and translation levels of STIM1 and Orai1 are regulated by multiple signaling pathways during tumorigenesis, progression, and metastasis in different organs. Moreover, STIM1 and Orai1 can be directly degraded by ubiquitination. Targeting these specific signaling molecules is a potential strategy for antitumor therapy.

## The functions of STIM1 in tumor cell death

Since the discovery of STIM1, its roles in platelet aggregation [52], allergic reactions [61], autoimmune diseases [62], tumor cell death [63], and T-cell differentiation [64] have been continuously investigated. Tumors are diseases characterized by an imbalance between cell proliferation and death. Cells die in disparate patterns in response to specific stimuli, and acquired defects in cell death signaling pathways are a feature of tumors. The aim of various antitumor treatments is to interfere with and block the process of proliferation and metastasis of tumor cells, thereby inducing the death of tumor cells. Therefore, it is crucial to clarify the mechanisms of tumor cell death in order to optimize antitumor treatment measures. Here, the tumor cell deaths regulated by STIM1 and their mechanisms are summarized (Table 1).

**Table 1:** Functions of STIM1 in tumor cell death.

Type of cell death	Cancer	The effect of STIM1/Ca^2+^	Reference
Apoptosis	Pancreatic cancer	Inhibit	[19]
	Gastric cancer	Inhibit	[68]
	Triple-negative breast cancer	Inhibit	[69]
	Head and neck squamous cell carcinoma	Inhibit	[70]
	Prostate cancer	Promote	[71]
Autophagy	Acute promyelocytic leukemia	Promote	[74–76]
	Gastric cancer	Promote	[77]
	Hepatocellular carcinoma	Promote	[78, 79]
	Prostate cancer	Promote	[80, 81]
Pyroptosis	Non-small cell lung cancer	Promote	[85]
	Colon cancer	Promote	[87]
Ferroptosis	Non-small cell lung cancer	Promote	[89]
	Pancreatic ductal adenocarcinoma	Promote	[91]

### STIM1 regulates tumor cell apoptosis

Apoptosis is a PCD whose basal function is to maintain tissue homeostasis [65]. Cancer is considered to be a disease of excessive cell proliferation, promoting apoptosis of tumor cells can effectively suppress tumor progression [66]. Ca^2+^ have been shown to regulate apoptosis in various tumors, whereas the mechanisms are elusive [67].

In most types of tumors, STIM1 attenuates apoptosis. For instance, in pancreatic cancer, STIM1 and Orai1 exert an inhibitory effect on apoptosis. Apoptosis of pancreatic cancer cells induced by chemotherapeutic agents such as 5-fluorouracil (5-FU) and gemcitabine can be enhanced by blocking STIM1 and Orai1 [19]. In gastric cancer, downregulation of STIM1 also promotes apoptosis [68]. Besides, Chakraborty’s study showed that phemindole could delay STIM1 translocation to PM in Triple-negative breast cancer (TNBC) cells, thereby reducing its binding to Orai1, causing ER stress and inducing apoptosis [69]. STIM1 is not only highly expressed in breast cancer, but also displays increased expression in 89% of head and neck squamous cell carcinoma (HNSCC). Li’s study presented that reducing intracellular Ca^2+^ concentration by suppressing STIM1 expression in HNSCC could accelerate ER stress-related cell death, such as apoptosis [70].

However, in prostate cancer (PCa), STIM1 plays an opposing role. Flourakis found that Orai1-induced Ca^2+^ entry is a major factor contributing to the apoptosis of PCa cells. Transfection of mutants R91W and L273S in PCa cells LNCaP, which impair Orai1 function, would reduce SOCE and thus protect LNCaP cells from TG-induced apoptosis [71].

In summary, targeting tumor cell apoptosis is a promising strategy for antitumor therapy. However, there is a large heterogeneity in the effects of STIM1 in different tumors, which may be related to their discrepant cell types and signaling pathways. Therefore, the molecular mechanisms of STIM1 regulation of apoptosis need to be investigated differentially for distinct tumor types.

### STIM1 promotes tumor cell autophagy

Autophagy is the process of isolating cytoplasm and organelles into autophagosome, which is transported to lysosome for proteolysis [72]. The essence of autophagy is to maintain the energy homeostasis of the cell and the organism [73]. Studies in the last decade have shown that STIM1 plays an important role in the induction of autophagy in tumor cells.

**Fig. 3:**
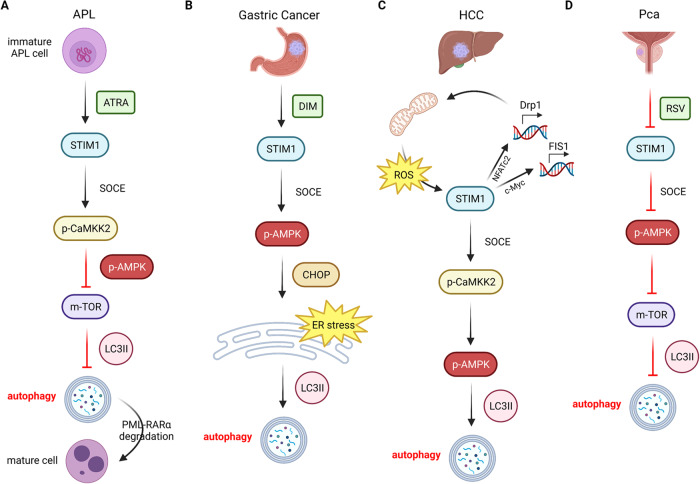
Schematic representation of the mechanism by which STIM1 regulates the AMPK signaling pathway through SOCE to induce autophagy in four types of tumor cells. **A** In APL, ATRA upregulates p-CaMKK2 and P-AMPK through activation of STIM1, which in turn inhibits mTOR expression, leading to autophagy. Subsequent degradation of PML-RARα oncoprotein allows APL to undergo granulocyte differentiation and thus transform into a mature state. **B** DIM is used to trigger autophagy in gastric cancer cells. The mechanism is that DIM raises STIM1-mediated SOCE, followed by upregulation of p-AMPK expression, and then induces ER stress via CHOP signaling. **C** In HCC, a positive feedback loop is formed between mitochondrial fission and the cytoplasmic Ca^2+^ signaling pathway. On the one hand, STIM1 induces autophagy by elevating the expression of p-CaMKK2 and p-AMPK; on the other hand, STIM1 promotes ROS production by mitochondrial fission through the transcriptional activation of Drp1 and FIS1 by NFATc2 and c-Myc, which in turn upregulates STIM1. **D** RSV has the ability to foster PCa autophagy, which, unlike other tumors, is dependent on blocking the expression of STIM1, thereby activates p-AMPK and inhibits the mTOR pathway.

For acute promyelocytic leukemia (APL), a non-solid tumor, all-trans retinoic acid (ATRA) is the first-line option for its treatment [74]. Previous studies have demonstrated that ATRA induces autophagy during granulocyte differentiation in APL patients, however, the upstream mechanism is unclear [75]. Merhi demonstrated that ATRA activates autophagy through the SOCE/calmodulin-dependent protein kinase kinase 2 (CAMKK2)/AMPK signaling axis, and these molecules could be potential targets to enhance the anti-cancer effects of ATRA [76] (Fig. 3A).

STIM1 can also regulate autophagy in solid tumors such as gastric cancer via AMPK. Ye found that 3,3’-Diindolylmethane (DIM) enhanced p-AMPK-CHOP-mediated ER stress through upregulation of STIM1-mediated SOCE, which in turn promoted autophagy in gastric cancer cells [77] (Fig. 3B). In addition, Huang et al. demonstrated that in HCC cells, the NF-κB pathway activated by increased reactive oxygen species (ROS) can upregulate STIM1, thereby activating SOCE function. The increased intracellular Ca^2+^ further promotes the expression of dynamin-related protein 1 (Drp1) and mitochondrial fission 1 (FIS1) via nuclear factor of activated T-cells 2 (NFATc2) and c-myc, thereby causing mitochondrial fission [78]. Furthermore, increased intracellular Ca^2+^ also stimulates the CaMKK/AMPK pathway, which leads to autophagy in HCC [79] (Fig. 3C). In contrast, Selvaraj’s experiments showed that resveratrol (RSV) activated AMPK by inhibiting STIM1, which in turn downregulated the V-akt murine thymoma viral oncogene homolog (AKT)/mammalian target of rapamycin (mTOR) signaling axis and ultimately promoted autophagy in PCa cells [80]. Furthermore, targeting the AKT-mTOR signaling pathway, Kondratskyi’s study elucidated that ML-9, an inhibitor of STIM1 and AKT, could promote autophagy in PCa cells by downregulating mTOR expression [81] (Fig. 3D). Besides, ML-9 has the effect of enhancing the anti-cancer activity of docetaxel, but its molecular mechanism remains to be investigated.

Taken together, the STIM1-SOCE-AMPK-autophagy pathway could be a potential therapeutic target for hematological cancers and solid tumors.

### Function of Ca^2+^ in other types of tumor cell deaths

The mechanisms by which STIM1 regulates apoptosis and autophagy in tumor cells have been more studied while the relationship between STIM1 and other types of tumor cell deaths (such as pyroptosis, ferroptosis, and cuproptosis) are still unclear. Recent studies have shown that calcium signaling can alter the levels of these PCDs in tumor cells, but whether STIM1 is involved requires further investigation.

#### Pyroptosis

Pyroptosis is caspase-1-mediated monocyte death characterized by the production of inflammasomes and caspase-11/4/5 is also activated during this process [82]. At present, the inflammasome sensors that have been extensively studied are nod-like receptor 1 (NLRP1), NLRP3, and nod-like nucleator 4 (NLRC4).

Cucurbitacin B (CuB) can induce apoptosis in tumor cells and has an inhibitory effect on the inflammatory response [83, 84]. Yuan et al. found that CuB binding to Toll-like receptor 4 (TLR4) promoted mitochondrial ROS production and intracellular Ca^2+^ accumulation through activation of the NLRP3 inflammasome complex, thereby facilitating non-small cell lung cancer (NSCLC) pyroptosis [85]. Selenoprotein has an important role in antioxidant and anti-inflammatory activities, and recent studies have shown that a certain amount of selenium intake also has a preventive effect on some tumors [86]. For example, Liu et al. showed that the selenoprotein receptor thioredoxin Reductase 3 (Txnrd3) expressed in the mouse colon and led to increased ER stress and Ca^2+^ release, which subsequently fostered the expression of NLRP3 and Gasdermin D (GSDMD) that could promote pyroptosis, thereby blunting the growth and proliferation of colon cancer cells [87].

#### Ferroptosis

Ferroptosis is a novel iron-dependent, lipid-reactive oxygen species (L-ROS)-induced PCD identified by Dixon in 2012 [88].

Chen et al. first found that erianin could restrain NSCLC cell proliferation and migration, and activate L-type voltage-dependent Ca^2+^ channel (LVDCC) by promoting the Ca/calmodulin (CaM) signaling pathway in NSCLC, while increasing Ca^2+^ transport and Fe^2+^ level, further promote the production of L-ROS and induce ferroptosis [89]. Mitochondria are essential for Ca^2+^ signaling and redox homeostasis [90]. Mitochondrial calcium uniporter (MCU) is an important complex that regulates Ca^2+^ homeostasis in mitochondria. MCU promotes cystine/glutamate antiporter SLC7A11 expression through activation of the Kelch-like ECH-associated protein 1-NF-E2-related factor 2 (KEAP1-NRF2) antioxidant pathway, which in turn promotes the migration, invasion and metabolic stress resistance in pancreatic ductal adenocarcinoma (PDAC) cells [91]. The relationship between the NRF2-dependent antioxidant system and the regulation of ferroptosis was also observed in melanoma. Wang et al. found that in melanoma, AMPK-mediated activation of CAMKK2 promoted NRF2 expression, thereby inhibiting L-ROS-dependent ferroptosis. Targeted inhibition of the AMPK-CAMKK2-NRF2 signaling axis could enhance the efficacy of anti-PD-1 antibody in melanoma [92]. However, whether and how STIM1 regulates ferroptosis await investigation.

#### Cuproptosis

Cuproptosis is the latest PCD discovered by Tsvetkov in 2022. Its mechanism is the direct binding of copper to the lipoylated components of the tricarboxylic acid (TCA) cycle, giving rise to the aggregation of lipoylated proteins, loss of iron-sulfur cluster proteins, and ultimately to proteotoxic stress and cell death [15]. The close association between Ca^2+^ with Cu^2+^ has been apprehended [93, 94], but whether Ca^2+^ is involved in the regulation of cuproptosis needs further study. Furthermore, their relationship between SOCE and cuproptosis remains unexplored.

## STIM1 mediates antitumor treatment resistance

Over the past 20 years, targeted therapies aiming at specific genes have been proposed in some cancers and the molecular mechanisms regulating cancer development have been identified, which has revolutionized antitumor treatment strategies. Nevertheless, despite significant advances in antitumor treatment, the inevitable drug resistance is a major obstacle to improving patient prognosis.

The evasion of apoptosis is one of the characteristics of tumor cells [95] and is a crucial reason for the low efficiency of treatment. Androgens are the standard treatment for early-stage PCa, but unfortunately, PCa progresses to an androgen-independent stage characterized by apoptosis resistance [96]. Flourakis’ team found in 2004 that apoptosis resistance in androgen-independent PCa cells was associated with SOCE downregulation [20]. Subsequently, in 2010, Flourakis demonstrated that the reduction in SOCE, caused by the downregulation of Orai1, led to the suppression of calcium-dependent apoptosis [76]. Dubois et al. found that overexpression of Orai3 in PCa cells disrupted the assimilation of Orai1, resulting in a reduction in SOCE density and causing apoptosis resistance [97]. Herein, targeting STIM1/Orai1/SOCE molecules is expected to reverse the apoptosis resistance of PCa cells. By contrast, in non-solid tumors such as B lymphoma, silencing of Orai1 enhanced the pro-apoptotic effect of rituximab. This suggests that the combination of rituximab with a CRAC channel inhibitor or Ca^2+^ inhibitor may improve the outcome of patients with B lymphoma [98].

In addition to endocrine and targeted therapies, tumor cell resistance to chemotherapy has been shown to be associated with the inhibition of apoptosis by STIM1. Sun et al. found significant upregulation of STIM1 in cisplatin-resistant osteosarcoma MG63 cells. Knockdown of STIM1 in MG63 cells increased the expression of GRP78, CHOP, and ATF4d, which induced ER stress and partially restored the sensitivity of cisplatin-resistant osteosarcoma cells [21]. 5-FU is also a commonly used chemotherapeutic agent in antitumor therapy and has the effect of inducing autophagy in tumor cells [99]. Tang’s study demonstrated that 5-FU caused autophagy of HepG2 cells by blocking the PI3K/AKT/mTOR signaling axis. Knockdown of Orai1 or using the SOCE inhibitor SKF96365 could further enhance the ability of 5-FU to inhibit this pathway, thereby sensitizing HepG2 cells to 5-FU treatment [100]. Therefore, in several tumors such as B lymphoma, osteosarcoma and HCC, STIM1 may protect them from escaping targeted therapy or chemotherapy. The use of Ca^2+^ or SOCE inhibitors in these tumors may partially restore their sensitivity to antitumor therapy.

## STIM1 in tumor immunity

Cancer consists of tumor parenchymal cells and the tumor microenvironment (TME). Enhancing the killing power of immune cells in TME can cause immunogenic cell death (ICD) of tumor parenchymal cells [18, 101]. Meanwhile, experiments in recent years have revealed that Ca^2+^ signaling is essential for lymphocyte function and that STIM1-deficient mammals develop immunodeficiency [102–104].

**Fig. 4:**
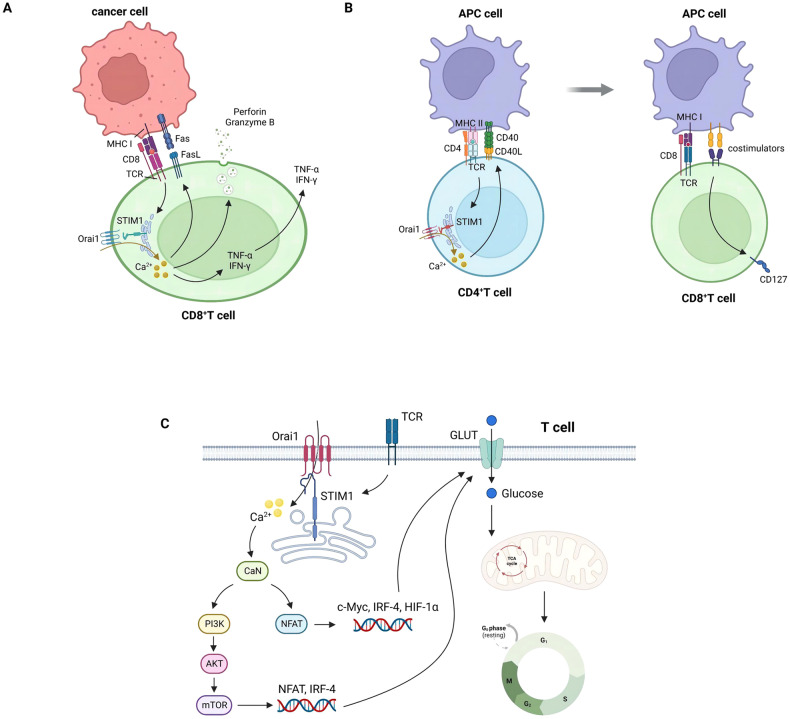
STIM1 enhances tumor killing, memory and proliferation of T cells. **A** Activation of STIM1 by TCR stimulation mediates the degranulation, production of TNF-α and IFN-γ and the expression of FasL by CD8^+^T cells, all of which are required for their antitumor immunity. **B** The memory response function of CD8^+^T cells (via expression of CD127) is dependent on CD40L expression in CD4^+^T cells facilitated by STIM1, which in turn accelerate antigen presentation by APCs. **C** STIM1 modulates T-cell proliferation by stimulating CaN to activate PI3K-AKT-mTOR and NFAT nutrient sensing pathways to elevate glucose uptake and entry into TCA utilization.

STIM1 and Orai1 are upregulated in T-cell-mediated immune responses and cause increased Ca^2+^ influx at the region where T cells make contact with the antigen-presenting DCs. Amplified Ca^2+^ signaling has a catalytic effect on T-cells’ activation, expansion and differentiation [105]. Weidinger found that STIM1 enhanced the cytotoxic effect of CD8^+^ T cells by increasing their FasL expression, exocytosis of perforin and granzyme B and the release of TNF-α and IFN-γ [106] (Fig. 4A). Furthermore, Shaw et al. demonstrated that STIM1 promoted CD40L expression on the surface of CD4^+^ T cells, thereby contributing to the memory response of CD8^+^ T cells upon reinfection [107] (Fig. 4B). In addition, STIM1 and calcineurin (CaN) synergistically activated the NFAT and PI3K-AKT-mTOR signaling pathways to stimulate naïve T-cell proliferation by promoting glucose uptake and utilization [108] (Fig. 4C).

T cells are the main effector cells of adaptive immunity, while neutrophils are also key cells in the innate immune response. Studies have shown that in STIM1-deficient mice, neutrophil degranulation, phagocytosis and ROS production were completely impaired, which correlated with reduced activation of protein kinase C isoforms α and β (PKC-α and PKC-β) leading to reduced phosphorylation of oxidase subunits [109]. Immediately afterward, Clemens et al. showed that STIM1 and STIM2 synergistically regulate SOCE function in neutrophils. However, STIM1 is the main factor affecting neutrophil bactericidal function, while STIM2 regulates the secretion of TNF-α, IL-10, and IFN-γ cytokines by neutrophils through activation of NF-κB [110].

Activation of cytotoxic T cells is dependent on the presentation of antigenic by DCs, thereby activating their adaptive immune responses to harmful stimuli. Studies by Nunes-Hasler have shown that STIM1 presents selective regulation of DCs. In myeloid STIM1-deficient mice, the phagocytosis and cross-presentation of antigens by DCs is reduced. However, STIM1 is not required for the upregulation of maturation markers in mouse myeloid-derived DCs, nor does it affect their differentiation [111]. Altogether, targeting STIM1 to enhance the cytotoxic effect of T cells on tumor cells is a promising therapeutic strategy.

## Therapeutic potential by targeting STIM1

The prognostic significance of STIM1 and its regulation of tumor cells has been demonstrated in a variety of cancers and is a hot topic of research in the field of oncology. As STIM1 is a fundamental component of the SOCE [112], compounds that block SOCE function can also restrain STIM1 to some extent. Over the last two decades, several SOCE inhibitors have been developed to bring hope for the antitumor treatment (Table 2).

**Table 2:** Drugs that are effective in therapies targeting STIM1.

Drug name	Disease	Mechanism
SKF96365	Breast cancer	Suppress tumor cell metastasis
	Cervical cancer	Suppress tumor cell growth
CM4620	Acute pancreatitis and COVID-19 pneumonia	Suppress inflammation
2-APB	Cervical cancer	Suppress tumor cell proliferation and metastasis
	Hepatocellular carcinoma	Suppress tumor cell proliferation
	Colon cancer	Suppress tumor cell metastasis
2-APB analogs	Still in the research phase	Inhibit the clustering of STIM1 and inactive the SOAR domain
Thapsigargin	Prostatic cancer	Suppress tumor cell growth
Optogenetic tools	Still in the research phase	Reconstruct the modular domains of STIM1 that enable SOCE activation

### SKF96365

SKF96365 was initially discovered as an inhibitor of ROC, but later studies revealed that it functions as an inhibitor of SOCE in a population of cells [113–115].

Yang et al. showed that STIM1 and Orai1 were involved in breast cancer metastasis. SKF96365 blocked STIM1 and Orai1-triggered SOCE and inhibited lung metastasis in mice with xenograft mouse model with MDA-MB-231 human breast tumor cells, suggesting that SKF96365 is a potential drug for antitumor metastasis [116]. Similarly, a study by Chen et al. found that silencing of STIM1 or use of SKF96365 reduced SOCE activity in cervical cancer SiHa cells and that tumor growth was inhibited by reduced vascularity and loss of blood supply to tumor cells in mice with subcutaneous tumor-bearing SiHa cervical cancer cells [117].

Notably, SKF96365 is not selective for CRAC channels and can also block other TRPCs [118]. Therefore, more studies on SKF96365 are needed to specifically delineate its mechanism in different types of tumor cells.

### CM4620

In comparison to the non-selective SOCE inhibitor SKF96365, CM4620 is a highly selective small-molecule inhibitor of the STIM1/Orai1 complex that effectively blocks CRAC [119]. For instance, a study conducted by Waldron et al. revealed that CM4620 reduced inflammatory features and cell death signaling in cases of acute pancreatitis [120]. Furthermore, clinical trials have demonstrated that Auxora, a novel intravenous CM4620 nanoemulsion formulation, can mitigate lung inflammatory signaling and protect lung tissue from calcium-mediated damage in patients with COVID-19 pneumonia [121]. In addition, CM4620 has also shown promise in alleviating allodynia in male mice, aside from its anti-inflammatory effects [120].

While CM4620 is not currently utilized for cancer treatment, its remarkable ability to selectively inhibit STIM1/Orai1-mediated SOCE makes it highly promising for therapeutic applications in tumors with upregulated STIM1/Orai1 expression.

### 2-APB

2-Diphenylboranyloxyethanamine (2-APB) has a dual effect, promoting store-operated calcium entry (SOCE) at low concentrations and inhibiting SOCE at high concentrations [122]. Studies have shown that 2-APB can suppress the proliferation of cervical cancer SiHa cells [117] and HCC Huh7 cells [123], as well as restrain the metastasis of cervical [124] and colon cancer cells [125].

To specifically block SOCE, two analogs of 2-APB, DPB-162AE, and DPB-163AE, were developed in 2010 [126]. These analogs effectively inactivate STIM1 by targeting the SOAR structural domain. Among them, DPB-162AE consistently inhibits SOCE regardless of the concentration [127]. However, further clinical trials are necessary to determine the safety and efficacy of DPB-162AE in human cancers.

### Thapsigargin

Tg is known for its high cytotoxicity and has been used in the treatment of rheumatic diseases, lung diseases, and female infertility [128]. Recent studies have revealed that Tg acts as a SOCE activator, inducing apoptosis by inhibiting the sarcoplasmic/endoplasmic reticulum Ca2+ ATPase (SERCA) pump [129]. To specifically target tumor cells, two prodrugs named G115 and G202 have been developed. These prodrugs are derived from Tg and are coupled to prostate-specific antigen (PSA). Currently, G115 and G202 are undergoing pre-clinical trials [130].

### Optogenetic tools

In recent years, there has been a significant increase in the use of optogenetics in various fields of basic medicine [131]. Ma et al. have made significant advancements by developing a set of genetically encoded Ca^2+^ actuators (GECAs) that effectively control the activation of store-operated calcium entry (SOCE), leading to precise modulation of intracellular Ca^2+^ concentrations [132]. However, it is important to note that optogenetics is currently primarily employed in neurological research and its potential impact on cancer therapy is still in the early stages of exploration.

## Conclusion and perspectives

In recent years, numerous studies have demonstrated the involvement of STIM1 in various aspects of cancer, including genesis, development, invasion, and metastasis. It exerts a significant influence on the fate of tumor cells. The regulation of STIM1 involves complex upstream mechanisms such as PI3K-SGK1-NF-κB, P300-HIF-1α, and miRNAs, which provide potential targets for precision antitumor therapy. Additionally, STIM1 has been found to impact tumor cell survival through various downstream signaling pathways. For instance, it promotes autophagy in different types of tumor cells via the SOCE-AMPK pathway, and enhances the self-renewal of HCC stem cells through the SOCE-NFATc2 axis. These signaling pathways hold promise for cancer intervention. However, it is important to note that the role of autophagy in cancer is paradoxical and dependent on the environmental conditions [133]. In the initial stages of tumorigenesis, autophagy plays a role in maintaining normal cellular physiology and metabolism. It enables lysosomes to break down damaged proteins and organelles, thereby exerting tumor-suppressive effects. However, as the tumor advances to a more advanced stage, nutrient scarcity and hypoxic conditions in the TME cause autophagy to support tumor survival and metastasis. Consequently, the choice to employ targeted therapies that either enhance or inhibit autophagy depends on the stage of the patient’s disease [134]. In addition, the role of STIM1 in apoptosis is relatively well understood compared to other forms of tumor cell death. However, most research has only focused on observing the phenomenon without delving into the underlying molecular mechanisms. As a result, the therapeutic targets for STIM1-induced cell death remain unclear.

To date, drugs such as SKF96365, CM4620, 2-APB, and their analogs have been studied as potential therapeutic agents for preventing tumor development by blocking STIM1 or SOCE. However, most of their effects have only been demonstrated in animal experiments, and further clinical trials are needed to assess their safety, efficacy, and determine the appropriate dosage for administration. In addition, tumor cells often exhibit resistance to various therapies, including chemotherapy, targeted therapy, immunotherapy, and endocrine therapy, due to their apoptosis resistance. This necessitates the use of STIM1/SOCE inhibitors to overcome immunotherapy resistance in lymphoma, chemotherapy resistance in osteosarcoma and HCC, and STIM1/SOCE activators such as thapsigargin to address androgen therapy resistance in PCa. However, it is important to note that the overall impact of non-specific STIM1 inhibition on antitumor therapy remains unknown, as STIM1 also plays a role in the activation and differentiation of CD8^+^ T cells, the secretory function of neutrophils, and the antigen-presenting role of DCs in immune cells within the TME. While the modulation of Ca^2+^ homeostasis through optogenetics is currently in the early stages of basic research, its potential applications in cardiac electrophysiology and neurology suggest that it could become a valuable tool for precision-targeted therapies. In summary, it is necessary to further investigate the specific mechanisms underlying different modes of tumor cell death. This will not only expand the range of targets for antitumor therapy but also improve the responsiveness to such therapy and enhance the killing effect of the TME. Ultimately, these advancements will lead to more effective antitumor treatments and greater health benefits for patients.

## Data Availability

All data are included in the manuscript.
